# Survival benefit with adjuvant chemotherapy in stage III microsatellite-high/deficient mismatch repair colon cancer: a systematic review and meta-analysis

**DOI:** 10.1038/s41598-022-05065-6

**Published:** 2022-01-20

**Authors:** Gianluca Tomasello, Michele Ghidini, Barbara Galassi, Francesco Grossi, Andrea Luciani, Fausto Petrelli

**Affiliations:** 1 Medical Oncology Unit, Fondazione IRCCS Ca’ Granda Ospedale Maggiore Policlinico, Via della Commenda 19, 20122 Milan, Italy; 2 Medical Oncology Unit, Medical Sciences Department, ASST Bergamo Ovest, Piazzale Ospedale 1, 24047 Treviglio, BG Italy

**Keywords:** Cancer, Gastrointestinal cancer, Colorectal cancer

## Abstract

Clinical observations have demonstrated that microsatellite instability-high (MSI-H) and/or deficient MMR (dMMR) status are associated with favorable prognosis and no benefit from 5-Fluorouracil (5-FU)-based adjuvant chemotherapy in patients with resected stage II colorectal cancer (CRC). This study represents a systematic review and meta-analysis exploring the predictive role of MSI-H status in stage III CRC undergoing or not adjuvant chemotherapy. Published articles that evaluated the role of adjuvant chemotherapy in resected stage III CRC from inception to September 2020 were identified by searching the PubMed, EMBASE, and Cochrane Library databases. The random-effects model was conducted to estimate the pooled effect size of OS and DFS. The primary outcome of interest was OS. 21,590 patients with MSI-H/dMMR stage III CRC, from n = 17 retrospective studies, were analyzed. Overall, OS was improved with any adjuvant chemotherapy vs. any control arm (single-agent 5-FU or surgery alone): HR 0.42, 95% CI 0.26–0.66; P < 0.01. Conversely, DFS was not significantly improved (HR 0.7, 95% CI 0.45–1.09; P = 0.11). In patients with stage III MSI-H/dMMR CRC, adjuvant chemotherapy is associated with a significant OS improvement. Thus, MSI-H/dMMR status does represent a predictive factor for postoperative chemotherapy benefit in stage III CRC beyond its prognostic role.

## Introduction

A large amount of evidence is currently available on the less beneficial, or even potentially detrimental effect of adjuvant, single-agent, fluoropyrimidine-based chemotherapy in patients with microsatellite high (MSI-H) or deficient mismatch repair (dMMR) stage II colorectal cancer (CRC)^1^. Much less clear is the impact of MSI-H/dMMR status in radically resected, node-positive CRCs. Updated results of the "MOSAIC" trial with 10-year median follow up, showed important disease-free (DFS) and overall survival (OS) improvements (hazard ratios [HRs] 0.48 [95% CI 0.20 to 1.12] and 0.41 [95% CI 0.16 to 1.07], respectively), in 9.4% of patients with stage II to III dMMR by the addition of oxaliplatin to 5-FU chemotherapy backbone^2^. However, the study resulted underpowered to show statistically significant differences due to the retrospective nature of the analysis and the small sample size. Further studies evaluating the effect of FOLFOX in patients with nonmetastatic MSI-H colorectal adenocarcinoma produced opposite results failing to find any correlation between MSI-H status and survival^3,4^. Therefore, we performed a systematic review and meta-analysis to explore the predictive role of MSI-H/dMMR status in stage III CRC patients undergoing or not adjuvant chemotherapy.

## Material and methods

### Search strategy and inclusion criteria

Three authors (GT, BG and FP) searched the databases PubMed, Cochrane Central Library, and Embase for potential articles published before September 2020. Search terms were: *(((("microsatellite instability"[All Fields] OR "MSI"[All Fields]) OR "dMMR"[All Fields]) AND (((((((((((((((("adjuvancy"[All Fields] OR "adjuvanted"[All Fields]) OR "adjuvanting"[All Fields]) OR "adjuvants"[All Fields]) OR "adjuvants pharmaceutic"[Pharmacological Action]) OR "adjuvants immunologic"[Pharmacological Action]) OR "adjuvants, pharmaceutic"[MeSH Terms]) OR ("adjuvants"[All Fields] AND "pharmaceutic"[All Fields])) OR "pharmaceutic adjuvants"[All Fields]) OR "adjuvant"[All Fields]) OR "adjuvants, immunologic"[MeSH Terms]) OR ("adjuvants"[All Fields] AND "immunologic"[All Fields])) OR "immunologic adjuvants"[All Fields]) OR "adjuvated"[All Fields]) OR "adjuvation"[All Fields]) OR "adjuvent"[All Fields]) AND (((((("chemotherapy s"[All Fields] OR "drug therapy"[MeSH Terms]) OR ("drug"[All Fields] AND "therapy"[All Fields])) OR "drug therapy"[All Fields]) OR "chemotherapies"[All Fields]) OR "drug therapy"[MeSH Subheading]) OR "chemotherapy"[All Fields]))) AND (((((("stage"[All Fields] OR "staged"[All Fields]) OR "stages"[All Fields]) OR "staging"[All Fields]) OR "stagings"[All Fields]) AND "III"[All Fields]) OR "Dukes C"[All Fields])) AND (((((((("colon"[MeSH Terms] OR "colon"[All Fields]) OR "colonic"[All Fields]) OR "colons"[All Fields]) OR "colon s"[All Fields]) OR "colonal"[All Fields]) OR "colonically"[All Fields]) OR "colonitis"[All Fields]) OR "colorectal"[All Fields]).*

Inclusion criteria were: (1) original articles (prospective or retrospective), with outcome data available, that compared the survival among adjuvant and no adjuvant chemotherapy arms in stage III MSI-H CRC, (2) comparison of single-agent 5-FU/capecitabine or combination chemotherapy with observation, (3) available hazard ratio (HR) for OS, disease-free survival (DFS), that compared experimental and control arms, or that may be calculated from survival curves. Papers were excluded if the number of patients in the MSI-H group was less than 10. We also did not include letters, review articles, and case reports. Data extraction was performed by two authors separately (FP and MG). If multiple articles were used to investigate the patients in the same clinical trial or medical institution, the latest or largest one would be included to prevent overlapping in case of disagreement, a third reviewer (FG) made the decision. The included studies included both randomized trials and retrospective series, therefore, both Cochrane and Newcastle–Ottawa scales were used to assess the methodological quality.

### Statistical analysis

The adjusted hazard ratios (HRs) calculated through a multivariate analysis were used to estimate the predictive value of MSI-H in stage III CRC. The HRs were extracted from the Cox proportional hazards regression model provided in the relevant articles. When the HR between experimental and control arms was not provided but the Kaplan–Meier survival curves were available, it was calculated with the method described by Tierney et al.^1^. Meta-analysis comparing treated and not treated patients aimed to explore the relationship between MSI-H status and benefit of adjuvant chemotherapy. RevMan 5.3 software was used for calculation. Heterogeneity was described using the I^2^ statistic. The random-effects model was used to estimate the effect size of OS and DFS. Funnel plots were provided in OS analysis to examine publication bias. Sensitivity analysis was also performed in OS analysis to describe heterogeneity. The RCT studies were assessed according to the Cochrane protocol^5^. The non-RCT studies were assessed using the Newcastle–Ottawa scale^6^, which considers participant selection, comparability, and outcome with a maximum total score of 9. A score higher than 7 was considered to be of good quality for the individual study.

## Results

**Figure 1:**
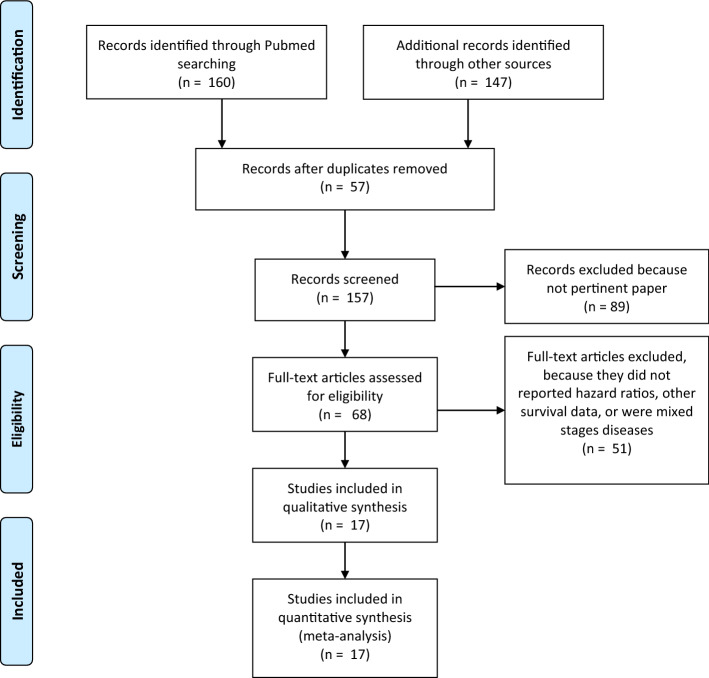
Flow diagram of included studies.

The flow of article selection process is depicted in Fig. 1. After screening 307 records, 17 studies finally met the predefined criteria and were considered eligible for inclusion in the systematic review^7–23^. Descriptive characteristics of the eligible studies are summarized in Table 1. There were n = 10 retrospective single-center series; n = 5 were analysis of n = 31 historical randomized trials, and 2 were cohort studies (n = 21,590 patients analysed). Patients were analyzed from 2004 to 2020. The median rate of MSI-H CRC was 13.7% (n = 2958). It was evaluated with immunohistochemistry biomarkers (n = 4), with MSI loci (n = 4) or both methods (n = 6). In 3 studies, method was not reported. Studies showed a prevalence of right sided CRC and of female patients. In 5 publications, experimental arms were combination chemotherapies (5-FU plus irinotecan or oxaliplatin or 5-FU + oxaliplatin + cetuximab). The quality of trials, was moderate (median NOS score 6.5) in retrospective studies with a low-moderate risk of bias in randomized trials.

**Table 1:** Characteristics of included studies.

Author/year	Type of publication	N° of pts	Median age	Sex m/f %/right CRC %	High risk features pt4/N2	IHC	Biomarkers	Follow up (months)	Schedule of adjuvant treatment (% received CT)	% of MSI pts	OS	DFS	Type of analysis	Quality (NOS or cochrane tool)
Alwers/2020^7^	Retrospective	461	–	–	–	MLH1-MSH2/6	Analysis of 3 markers	74.4	5-FU or 5-FU + OXA (77%)	15.8	Yes	Yes (RFS)	MVA	8
Chouhan/2018^8^	Retrospective	686	–	–	–	–	≥ 2 markers/10	52	Single agent 5-FU (33.6%)	13.8	Yes (CSS)	No	MVA	7
DE VOS TOT NEDERVEEN CAPPEL/2004^9^	Cohort study	92	45.5	58/42/–	–	–	–	48	Single agent 5-FU (30.4%)	100	Yes (CSS)	Yes	UVA	6
Elsaleh/2011^10^	Cohort study	876	70	35/65/92	–	–	–	76	Single qgent 5-FU (33%)	7.8	Yes	No	MVA	8
Klingbiel/2015^11^	Retrospective analysis of PETACC-3 trial	859	54	–/76	18.9/13	–	≥ 3 markers/5	69.1	5-FU + AF vs FOLFIRI (100%)	12.1	Yes	Yes (RFS)	MVA	Moderate
Lanza/2006^12^	Retrospective	325	–	–	–	MLH1-MSH2	–	93.9	5-FU-based (21.9%)	12.6	Yes (CSS)	No	UVA	8
Ogino/2012^13^	Retrospective analysis of CALGB 89,803 trial	506	–	–	–	MLH1-MSH2	≥ 5 markers/10	91.2	5-FU vs IFL (100%)	15	Yes	Yes (DFS)	MVA	Low
Ooki/2014^14^	Retrospective	405	–	32/68/85	12.5/20	–	≥ 2 markers/5	57.3	5-FU or 5-FU + OXA (60%)	2.4	Yes	Yes (DFS)	UVA	7
Sasaki/2016^15^	Retrospective analysis of 2 RCTs	300	–	–	–	MLH1-MSH2/6	–	74.8	UFT (50%) vs surgery	8	Yes	No	UVA	Low
Shaib/2020^16^	Retrospective	9226	65	45/55/67	–	–	–	–	Single agent 5-FU or polychemotherapy (87.7%)	25.8	Yes	No	MVA	6
Sinicrope/2011^17^	Retrospective analysis of n = 25 RCTs	1363	–	–	–	IHC or > 30% of markers out of 5 MSI loci		96	Single agent-5FU or polychemotherapy or portal infusion 5FU* (50%) vs surgery or no-5-FU	15.2	Yes	Yes (DFS)	MVA	Moderate
Tan/2018^18^	Retrospective	299	65	41/59/52	–	MLH1-MSH2/6	–	32	5-FU or 5-FU + OXA (67.8%)	9	Yes (CSS)	No	MVA	6
Thomas/2015^19^	Retrospective	814	70	50/50/49	–/28.9	–	≥ 2 markers/5	36.3	5-FU (35%)	9.4	Yes (CSS)	No	MVA	6
Tougeron/2016^20^	Retrospective	185	–	–	–/16.2	MLH1-MSH2/6	≥ 2 markers/5	47	5-FU or 5-FU + OXA (68.9%)	61.9	No	Yes (DFS)	MVA	6
Wang/2019^21^	Retrospective	286	–	50/49/69	–	MLH1-MSH2	≥ 2 markers/5	56	CAPOX or FOLFOX (75%)	19.2	Yes	No	MVA	7
Zaanan/2010^22^	Retrospective	233	59	47/53/75	–/54.4	MLH1-MSH2/6	–	46	5-FU or FOLFOX (100)	13.7	No	Yes (DFS)	UVA	6
Zaanan/2020^23^	Retrospective analysis of 2 RCTs	4674	56	50/50/85	19/42	MLH1-MSH2/6	≥ 3 markers/5	50.4	FOLFOX ± cetuximab (100%)	10.6	No	Yes (DFS)	MVA	Low

*Not containing oxaliplatin or 5FU, °All stage III patients, *IFL* irinotecan + bolus 5FU + bolus leucovorin, *AF* folinic acid, *UFT* uracil + ftorafur, *CSS* cancer-specific survival, *M* male, *F* female, *CRC* colorectal cancer, *pts* patients, *UVA* univariate analysis, *MVA* multivariate analysis, *NOS* Nottingham-Ottawa scale, *OS* overall survival, *DFS* disease-free survival, *MSI* microsatellite instability, − not available or not reported.

**Figure 2:**
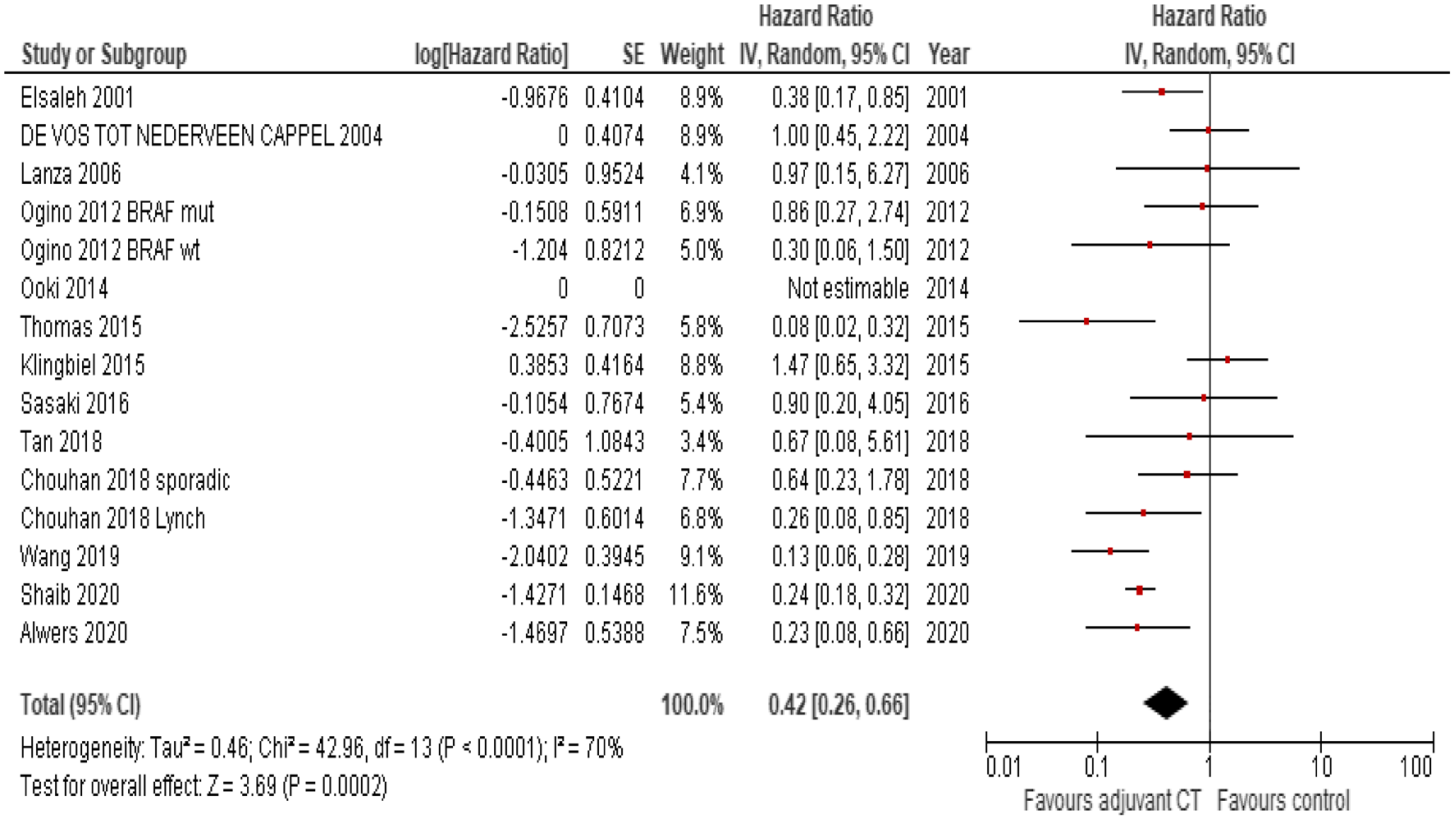
Forest plot for overall survival in MSI-H treated with adjuvant chemotherapy (polychemotherapy or single agent) vs control arms (single agent or surgery alone).

**Figure 3:**
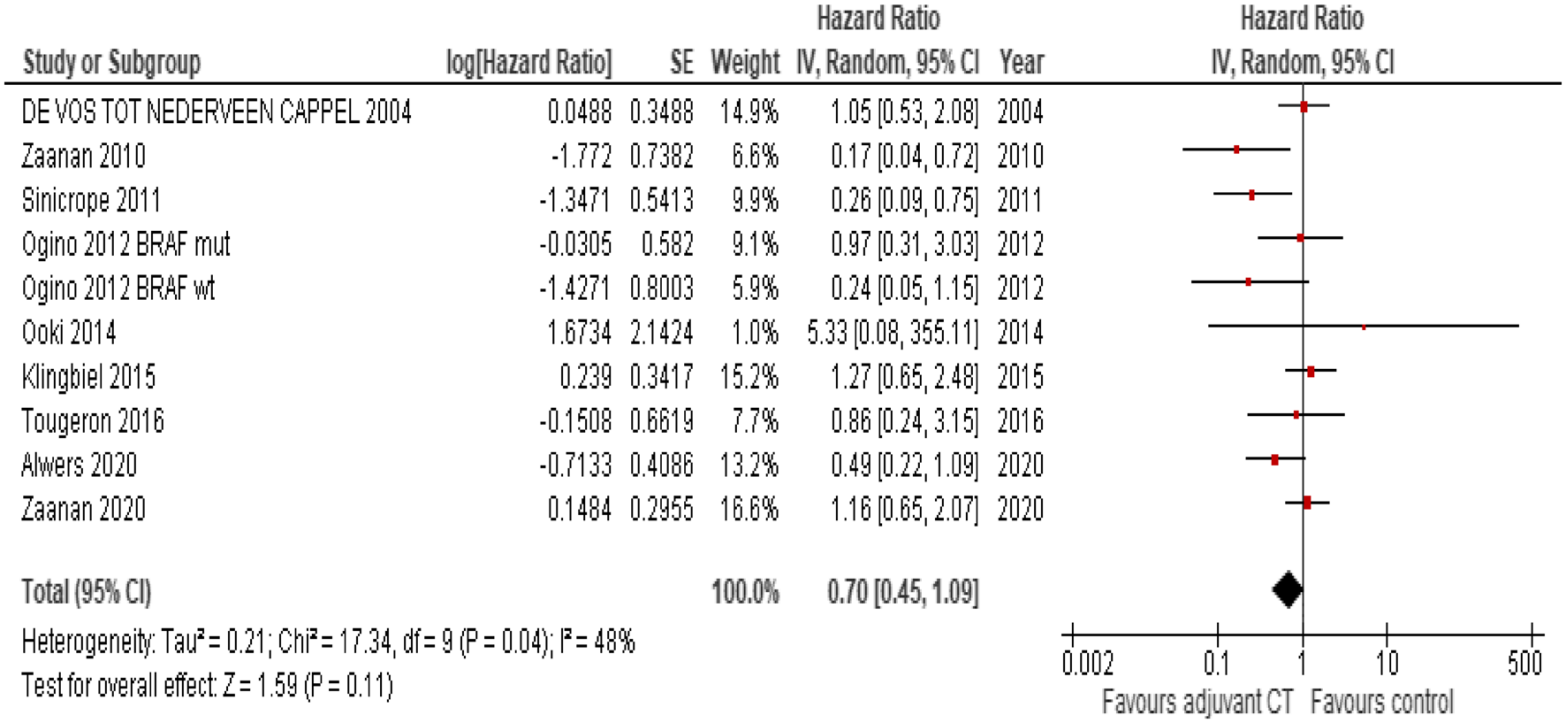
Forest plot for disease-free survival in MSI-H treated with adjuvant chemotherapy (polychemotherapy or single agent) vs control arms (single agent or surgery alone).

Overall survival was improved with any comparison of any adjuvant chemotherapy vs any control arm (HR 0.42, 95% CI 0.26–0.66; P < 0.01; Fig. 2). Conversely, DFS was not significantly improved (HR 0.7, 95% CI 0.45–1.09; P = 0.11; Fig. 3). In studies where any type of adjuvant chemotherapy (combination chemotherapy or single agents) was compared to surgery alone, survival was even more improved (HR for OS 0.34, 95% CI 0.22–0.54; P < 0.01). In studies where polychemotherapy was the experimental arm the benefit was of similar magnitude (HR 0.37, 95% CI 0.19–0.74). The results were significant in retrospective series (HR for OS 0.32, 95% CI 0.20–0.51; P < 0.01) but not in n = 3 randomized studies (HR 0.98, 95% CI 0.55–1.75; P = 0.94). However, this last analysis included only 204 patients.

To explore if clinical risk classification (defined high risk when tumor had pT4 and/or pN2 status) may influence benefit of adjuvant chemotherapy we performed a meta-regression analysis according to low-risk population. This analysis was possible only for DFS analysis for lack of sufficient data in OS population and confirmed a significant trend for reduced DFS benefit for adjuvant chemotherapy in low risk subgroup (P = 0.04).

**Figure 4:**
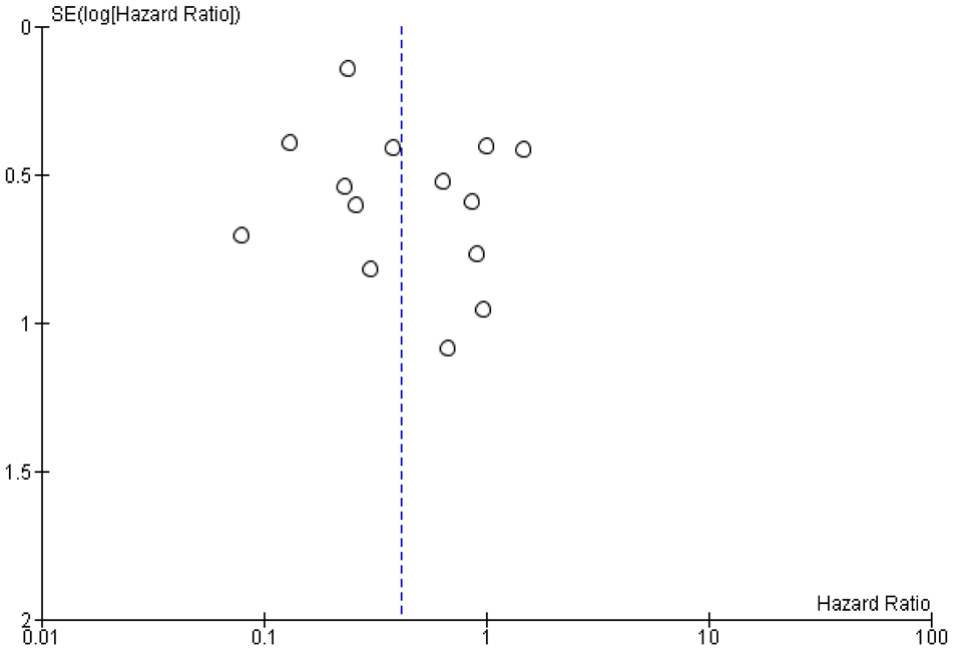
Funnel plot for publication bias for overall survival.

Begg's and Egger's tests were not significant for publication bias (P = 0.31 and P = 0.08; Fig. 4)^24^.

## Discussion

Stage II colorectal tumors harboring microsatellite instability or dMMR status are commonly associated with a more favorable prognosis as compared to their MSS counterpart. Patients in the same stage without high-risk features are also not likely to derive significant benefit from adjuvant fluoropyrimidine-based therapy. While the negative impact of adjuvant chemotherapy in stage II has been established, less clear is the role of postoperative treatments for MSI-H/dMMR stage III CRC. Unfortunately, little evidence is currently available for this patient subgroup, coming almost exclusively from retrospective analyses of large randomized controlled trials or single-center case series. Most early studies failed in demonstrating any survival benefit associated with fluoropyrimidine chemotherapy. However, a recently published large multicenter AGEO study indicated opposite results^20^. Specifically, this retrospective trial included 433 dMMR stage II/III CRC patients who received surgery alone (n = 263) or surgery plus adjuvant chemotherapy consisting of fluoropyrimidine with (n = 119) or without (n = 51) oxaliplatin. Interestingly, at multivariate analysis, a statistically significant DFS advantage (HR 0.41, 95% CI 0.19–0.87, *P* = 0.02) in favor of stage III patients receiving adjuvant fluoropyrimidine plus oxaliplatin chemotherapy (n = 89) compared with those treated with surgery alone (n = 58) was shown, while chemotherapy with fluoropyrimidine alone (n = 40) was not associated with a longer DFS compared with surgery alone (HR 0.66, 95% CI 0.29–1.50, *P* = 0.32).

Despite the clear limitation of the nonrandomized and retrospective design of all studies included, this meta-analysis provides evidence that adjuvant chemotherapy is beneficial in patients with stage III MSI-H/dMMR colorectal cancer. Specifically, in patients receiving any adjuvant chemotherapy, the risk of death is reduced by 58% compared with surgery alone or 5-FU single-agent. More interestingly, compared with only observation following surgery, mono- or combination chemotherapy was associated with a more considerable OS benefit corresponding to an impressive death risk reduction by 66%.

Although a DFS difference between any adjuvant treatment and surgery was evident, it did not reach statistical significance. This finding may be explained with the fact that some large cohort or registry-based series collected only survival data, with DFS available in only half of studies. Also, MSI-H/dMMR are low rate of relapse events, in particular for N1 sub stage, and a prolonged post recurrence survival as showed in the ACCENT analysis previously published by Tajeb et al.^25^.

Our analysis has some intrinsic limitations. Firstly, despite no obvious publication bias, we observed notable heterogeneity due to retrospective nature of the study, and the inclusion of relatively different populations. We took this into account with a random effect model analysis and with subgroup analysis. In addition, a significant difference was observed for the type of MMR evaluation (biomarker vs. IHC analysis) and the quality/size of publications. Secondly, this meta-analysis was based on published data instead of individual patient data. Thirdly, analysis was not performed according to side (right vs. left CRC), nodal stage (high vs. low-risk stage III disease), and for colon vs. rectal cancers. Fourth, we did not consider the prognostic effect of MSI status, but this was already known from previous reviews and meta-analysis of randomized studies that showed a similar prognosis of MSS, stage III tumors. However, this represents the more comprehensive and updated meta-analysis exploring the predictive effect of MSI status in stage III CRC.

Our results align with those very recently published from an ACCENT pooled analysis of twelve adjuvant trials^26^, which evaluated the effect of fluoropyrimidine with or without oxaliplatin on DFS and OS on a large cohort of patients with MSI stage III CRC. This study showed that the combination of fluoropyrimidine plus oxaliplatin significantly improves OS of patients with MSI/dMMR stage III CRC (HR, 0.52; 95% CI, 0.28 to 0.93) from the two randomized trials testing fluoropyrimidines with or without oxaliplatin. Moreover, authors investigated the prognostic value of MSI among the 4,250 patients treated with fluoropyrimidines plus oxaliplatin, and found that MSI was associated with better OS in the N1 group compared with MSS (adjusted HR 0.66; 95% CI 0.46–0.95) but similar survival in the N2 population (adjusted HR 1.13; 95% CI 0.86–1.48; *P* interaction = 0.029). Our analysis provide a more expanded population of MSI CRC patients including also n = 31 randomized studies other than observational series (n = 2958), and showed a trend for better DFS with adjuvant chemotherapy in high-risk stage III CRCs. Also, unlike ACCENT analysis where no significant benefit was registered with 5FU alone, in the present cohort we found a similar benefit of adjuvant chemotherapy independent of treatment schedule.

In conclusion, based on the findings of our meta-analysis, MSI-H/dMMR condition should be regarded as an important predictive factor for adjuvant chemotherapy benefit in node-positive radically resected CRC patients, in particular with high risk features (pT4 and/or pN2 disease). According to age and performance status, when feasible, and in the absence of specific medical contraindications to chemotherapy, at least single-agent 5-FU or combination treatments should be recommended. Studies exploring the addition of immune-checkpoint inhibitors to chemotherapy in this specific setting are currently ongoing. Hopefully, positive results will further improve and expand the available therapeutic options.
